# Real-world evidence for the effectiveness and safety of dupilumab in patients with CRSwNP after 1 year of therapy

**DOI:** 10.1016/j.waojou.2023.100780

**Published:** 2023-05-18

**Authors:** Tobias Albrecht, Martin M. Sailer, Flavia Capitani, Carolina van Schaik, Hubert Löwenheim, Sven Becker

**Affiliations:** a Department of Otorhinolaryngology, Head and Neck Surgery, University Hospital Tübingen, Germany; b Otorhinolaryngology Medical Center, Dres. Sailer, Göppingen, Germany

**Keywords:** CRSwNP, Real-world evidence, Nasal polyps, Sinusitis, Dupilumab, Antibodies, Monoclonal, Quality of life, Eosinophils

## Abstract

**Objectives:**

For nearly 3 years, the monoclonal antibody dupilumab has been approved in Germany for the treatment of patients with chronic rhinosinusitis with nasal polyps (CRSwNP). Although efficacy has been demonstrated in large double-blind, placebo-controlled clinical trials, few reports of real-world data on this therapy have been published to date.

**Methods:**

Patients with an indication for treatment with dupilumab for CRSwNP were included in the study and followed up every 3 months for a period of 1 year. At the baseline visit, demographic data, medical history, comorbidities, nasal polyp score, disease-related quality of life (SNOT-22), nasal congestion, and sense of smell (VAS and Sniffiń Sticks test) were recorded. In addition, total blood eosinophil counts and serum total IgE were measured. During follow-up, all of the described parameters and possible adverse events were recorded.

**Results:**

Eighty-one patients were enrolled in the study, of whom 68 patients were still receiving dupilumab after 1 year of follow-up. Eight patients discontinued therapy, with only 1 patient discontinuing due to severe side effects. The Polyp score decreased substantially during follow-up, and parameters for disease-related quality of life and sense of smell increased significantly. Total IgE levels decreased significantly, and eosinophils leveled off at baseline after an initial increase after three months of therapy. No clinical data could be identified to a priori predict a treatment response.

**Conclusions:**

Dupilumab shows effectiveness and safety in the treatment of CRSwNP under real-world conditions. More research on systemic biomarkers and clinical parameters to predict treatment response is necessary.

## Introduction

Chronic rhinosinusitis (CRS) is one of the most common chronic diseases of the upper respiratory tract with an extremely heterogeneous clinical presentation and a high socioeconomic impact.^1^^,^^2^ Clinically, CRS is divided into 2 phenotypes, CRS with nasal polyposis (CRSwNP) and CRS without nasal polyposis (CRSsNP). The increasing understanding of the pathomechanisms of CRS in recent years has led to a classification into 3 primary endotypes of inflammation usually associated with physiologic immune responses across mucosal boundaries. Type 1 responses are physiologically directed against intracellular pathogens, primarily viruses, and are characterized by elevated levels of IFN-γ. Type 2 responses are directed against large extracellular parasites. IL-17 and IL-22 are increased in type 3 reactions, which are directed against extracellular bacteria and fungi. In CRS, a chronic activation of 1 or more of these signaling pathways can be found in sinus tissue.^3^ In approximately 80% of caucasian patients with CRSwNP the disease is caused by a type-2 inflammation.^1^^,^^4^ A Type 2 inflammation is characterized by an elevation of cytokines, such as interleukin (IL)-4, IL-5 and IL-13, and an activation and recruitment of eosinophils and mast cells. Patients with a type 2 endotype tend to be much more resistant to traditional therapies, exhibiting a high recurrence rate when compared with pure type 1 or 3 endotypes.^3^

In November 2019, German health authorities approved the first monoclonal antibody, which interferes with the inflammatory pathway of type 2 inflammation, for the treatment of CRSwNP.^5^ Dupilumab is a monoclonal antibody that binds to the alpha subunit of the IL-4 receptor and thereby blocks signaling of the IL-4 and IL-13 pathways.^6^

Phase III trials have described substantial improvement in patients' symptoms under antibody therapy.^7^, ^8^, ^9^, ^10^ The use of antibodies represents a milestone in the treatment of this disease, which in recent decades could only be treated with topical and/or systemic corticosteroids, nasal irrigation, and repetitive functional endoscopic sinus surgery in cases of recurrent disease.

Phase III trials that lead to the approval of this antibody therapy provide robust evidence for its clinical efficacy and safety. However, the internal validity attained in these trials is often achieved at the expense of uncertainty about generalizability, especially since the populations enrolled in such studies may differ in significant ways from those seen in practice.^11^ The aim of this real-world observational study was to investigate the effectiveness and possible side effects of the monoclonal antibody dupilumab in a cohort of patients treated for one year under real-life conditions according to the recommendations of the European Position Paper on Rhinosinusitis and Nasal Polyps 2020 (EPOS 2020) for therapy with biologics.^3^

### Material and methods

The research protocol for this prospective study was approved by the institutional ethics committee (University of Tübingen #873/2018BO2). The study population included patients with CRSwNP who underwent therapy with the monoclonal antibody dupilumab in the Department of Otorhinolaryngology and Head and Neck Surgery at the University Medical Center of University of Tübingen, Germany and were able to give informed consent prior to the planned therapy. Patients were treated with 300 mg of subcutaneous dupilumab every 2 weeks as an add-on therapy to intranasal corticosteroids according to the drug approval regulations in Germany. Demographic characteristics were recorded at study enrollment and patients were followed up every 3 months for a total period of 1 year. The decision for treatment with the monoclonal antibody was in accordance with the current EPOS 2020 recommendations.^3^ The SNOT-22 questionnaires on quality of life^12^ and symptom control, as well as a self-assessment of nasal congestion using a visual analog scale (VAS), were answered by the patients. If the patients had comorbid asthma, the GINA score was evaluated.^13^ Subjective impairment due to loss of smell was additionally evaluated by a VAS. In addition to the questionnaires, a clinical examination was conducted at every timepoint, and the bilateral endoscopic nasal polyp score (NPS) was recorded.^14^ The NPS was scored by 2 independent investigators at each time point. Every visit included an odor test with the 12-item Sniffin’ sticks test.^15^ Furthermore, total blood eosinophils and total IgE in serum were determined.

Statistical analysis for paired data was performed using Student's *t*-test for parametric data and Wilcoxon matches pairs single rank test for nonparametric data. For unpaired tests, Mann-Whitney test was performed. P values < 0.05 were accepted to indicate statistical significance. Unless stated otherwise, data are presented as the means with standard deviations.

## Results

### Clinical characteristics of patients

During the observation period, 81 patients with uncontrolled CRSwNP were treated. From initiation of the treatment, patients were followed up for 1 year. After 1 year of follow up, 68 (83.95%) of the initial 81 patients were still receiving dupilumab. Eight patients (9.9%) discontinued therapy with dupilumab because of side effects, 1 patient due to severe side effects. In 5 patients, therapy was switched to another biologic due to an inadequate response to therapy.

The demographic characteristics and clinical baseline values obtained from the complete cohort as well as the characteristics of the subgroups of patients who were treated the whole year with dupilumab and the subgroup that stopped therapy are shown in Table 1. No significant differences were found between the two subgroups in terms of clinical or demographic characteristics.

**Table 1:** Descriptive characteristics of the total study population (n = 81) as well as the subgroups “completed”, with no change of therapy for one year (n = 68) and “therapy changed” (n = 13). Data shown as mean value ± standard deviation and percentages in brackets

	Total (n = 81)	Completed (n = 68)	Therapy Change/Stop (n = 13)
Age – yr	49.72 ± 14,2	49.81 ± 12.58	46.46 ± 18.4
Sex, Female, n (%)	39 (48.15%)	32 (47.06%)	7 (53.85%)
Time since most recent polyp surgery (y)	4.44 ± 3.9	4.39 ± 3.65	4.85 ± 5.65
Nasal-Polyp surgery			
≥ 1 previous surgery	81 (100%)	68 (100%)	13 (100%)
≥ 3 previous surgery	52 (64.2%)	45 (66.18%)	7 (53.85%)
≥ 5 previous surgery	11 (13.6%)	9 (13.24%)	2 (15.38%)
Systemic corticosteroid in the last 2	60 (74.1%)	49 (72.1%)	11 (84.6%)
Aspirin desensitization	11 (13.58%)	9 (13.24%)	2 (15.38%)
SNOT-22 total score before therapy	55.04 ± 16.9	53.74 ± 17.62	60.38 ± 11.17
VAS nasal congestion before therapy (scale 0–10)	6.36 ± 2.6	6.32 ± 2.66	6.28 ± 2.25
GINA Score in patients with comorbid asthma before therapy	2.7 ± 1.07	2.71 ± 1.07	2.63 ± 1.19
NPS before therapy (scale 0–8)	5.31 ± 1.87	5.44 ± 1.79	4.62 ± 2.18
Smell-VAS initial	9.14 ± 1.79	9.16 ± 1.85	9.03 ± 1.51
Smell Test Score (Sniffin’ sticks screening Test, Scale 0–12)	2.47 ± 2.79	2.26 ± 2.72	2.85 ± 3.05
Baseline Blood IgE (IU/ml)	267.19 ± 405.64	251.43 ± 339.46	260.81 ± 741.37
Baseline Blood eosinophils (n/μl)	472.49 ± 401.15	475.51 ± 397.39	456.92 ± 436.51
Other biologicals before treatment with dupilumab	3 (3.7%)	3 (4.41%)	0 (0%)
Comorbidity, n (%)	78 (96.3%)	67 (98.53%)	11 (84.62%)
Non	3 (3.7%)	1 (1.47%)	2 (15.38%)
Allergic Rhinitis intermittent	24 (29.63%)	19 (27.94%)	5 (38.46%)
Allergic Rhinitis persistent	18 (22.22%)	18 (26.47%)	0 (0%)
Other Allergy (Food/Medication)	19 (23.46%)	14 (20.59%)	5 (38.46%)
NSAID intolerance	36 (44.44%)	30 (44.12%)	6 (46.15%)
Asthma	61 (75.31%)	53 (77.94%)	8 (61.54%)

### Evaluation of clinical parameters

#### SNOT-22 questionnaire

The SNOT-22 questionnaire consists of 22 questions about rhinosinusitis-specific symptoms and health-related quality of life (HRQL). Patients were asked to answer the questions at each time point of data acquisition with reference to the past 14 days, considering both the severity of symptoms and the frequency of occurrence. A change in the score of ≥8.9 points is classified as a minimal clinically relevant change (MCID).^16^

**Fig. 1:**
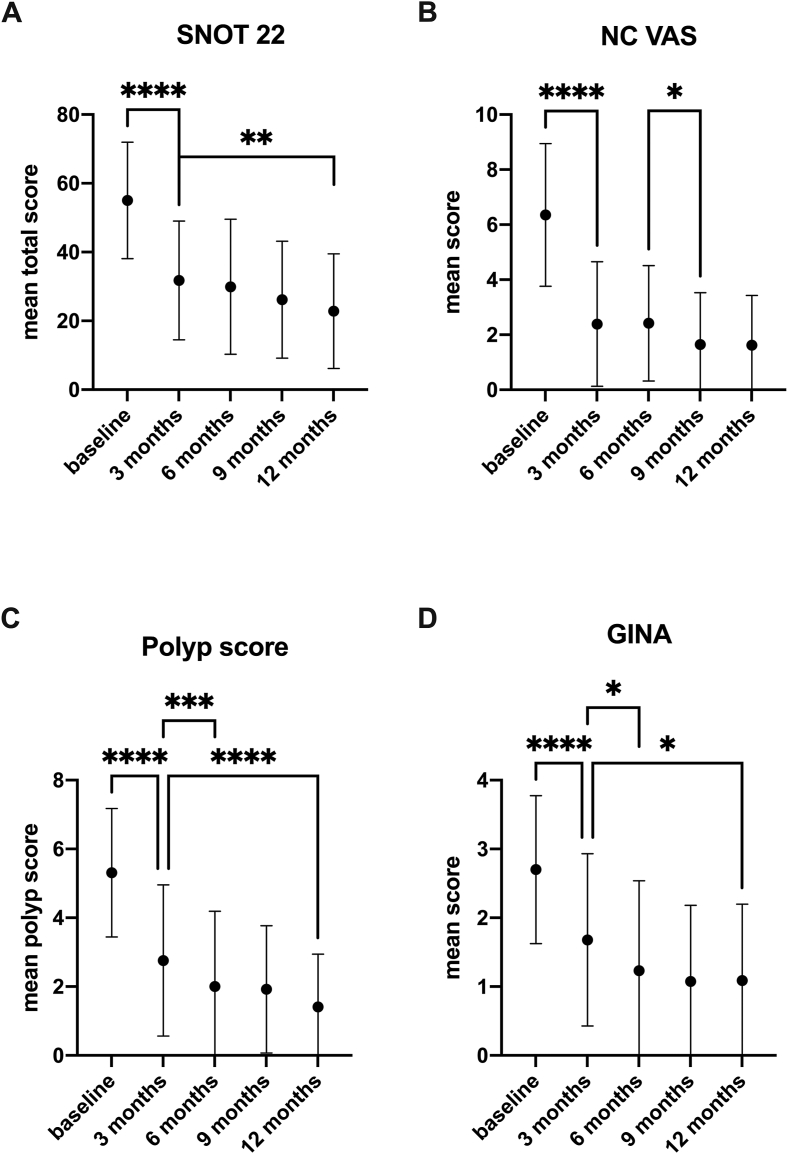
Values collected before, 3,6,9 and 12 months after initiation of dupilumab therapy. **A**): health-related quality of life measured with the SNOT-22. Mean total scores (dots) with standard deviation. ∗∗∗∗p < 0.0001; ∗∗p < 0.005. **B**) Symptom severity of nasal congestion on a VAS (scale from 0 to 10). Mean values (dots) with standard deviation. ∗∗∗∗p < 0.0001; ∗p < 0.05. **C**) total nasal polyp score. Mean values (dots) with standard deviation. ∗∗∗∗p < 0.0001; ∗∗∗p = 0.001. **D**) GINA scores in individuals with comorbid asthma. Mean values (dots) with standard deviation. ∗∗∗∗p < 0.0001; ∗p < 0.05.

Significant improvements occurred as early as the first assessment timepoint after treatment initiation, with a continuous improvement up to the end of the observation period. The total SNOT-22 score decreased significantly from a baseline value of 55.04 ± 16.9 to 31.78 ± 17.27 after 3 months of therapy (p < 0.0001; −23.25 ± 2.714 SEM) and continued to decrease to a total score of 22.85 ± 16.66 at 12 months of therapy. After 3 months 79.49% of the patients had a clinically meaningful improvement in total SNOT-22 scores. The proportion among patients with a clinically meaningful improvement increased to 89.1% after 12 months of treatment. The course of the SNOT-22 total values over the survey period is shown in Fig. 1A.

#### Nasal congestion VAS

Nasal congestion is a common symptom in patients with CRS and has a notable impact on quality of life, emotional function, productivity, and the ability to perform daily activities.^17^ Patients were asked to document the intensity of the nasal congestion on a visual analog scale from 0 to 10 when presenting at the clinic. Nasal congestion improved the most during the first 3 months of therapy from a score of 6.36 ± 2.6 to 2.39 ± 2.26 and continued to improve slightly to 1.62 ± 1.8 after 12 months of therapy. The VAS scores at the assessed timepoints are shown in Fig. 1B.

#### NPS

The NPS is used to assess the size of the nasal polyps. For each nostril, the size of the polyps is scored on a scale of 0 (no polyps) to 4 (complete blockage of the nasal cavity). Scores of each nostril are combined to form the bilateral NPS, which accordingly can take values from 0 to 8. The mean combined NPS of the observed population was 5.31 ± 1.87 at the pretreated timepoint and decreased significantly to 2.76 ± 2.19 (p < <0.0001) after 3 months of therapy. The NPS continued to decrease to 1.41 ± 1.54 at 12 months of therapy. After 3 months 87.34% of the patients improved by ≥1 in the NPS, and 68.35% even improved by ≥2 points. After 12 months of therapy 98.21% of the treated population improved by ≥1 point and 87.5% by ≥2. The NPS values at the assessed timepoints are shown in Fig. 1C.

#### Loss of smell assessed by Sniffin’ Sticks and symptom severity assessed by VAS

The Sniffin’ Sticks test is an olfactory identification test consisting of 12 different everyday odors. Each odorant is accompanied by a multiple-choice question with four alternative response options to describe the odor, from which the patient selects 1 answer. This results in a score of 0–12 possible correct answers, whereby a lower score corresponds to a more severe impairment of olfaction.^18^

In addition to the measurement of olfaction with the Sniffin’ Sticks test, the patients were asked to document their personal perceived limitation in regards to their olfaction on a VAS from 0 to 10, where 10 indicated the most severe restriction imaginable.

Before treatment, the perceived impairment by the loss of smell was rated 9.14 ± 1.79. After 3 months of treatment, patients’ perceived restriction significantly improved to 4.56 ± 3.19 (p < 0.0001) and continued to improve to 3.09 ± 2.73 (p = 0.0005) after 12 months.

**Fig. 2:**
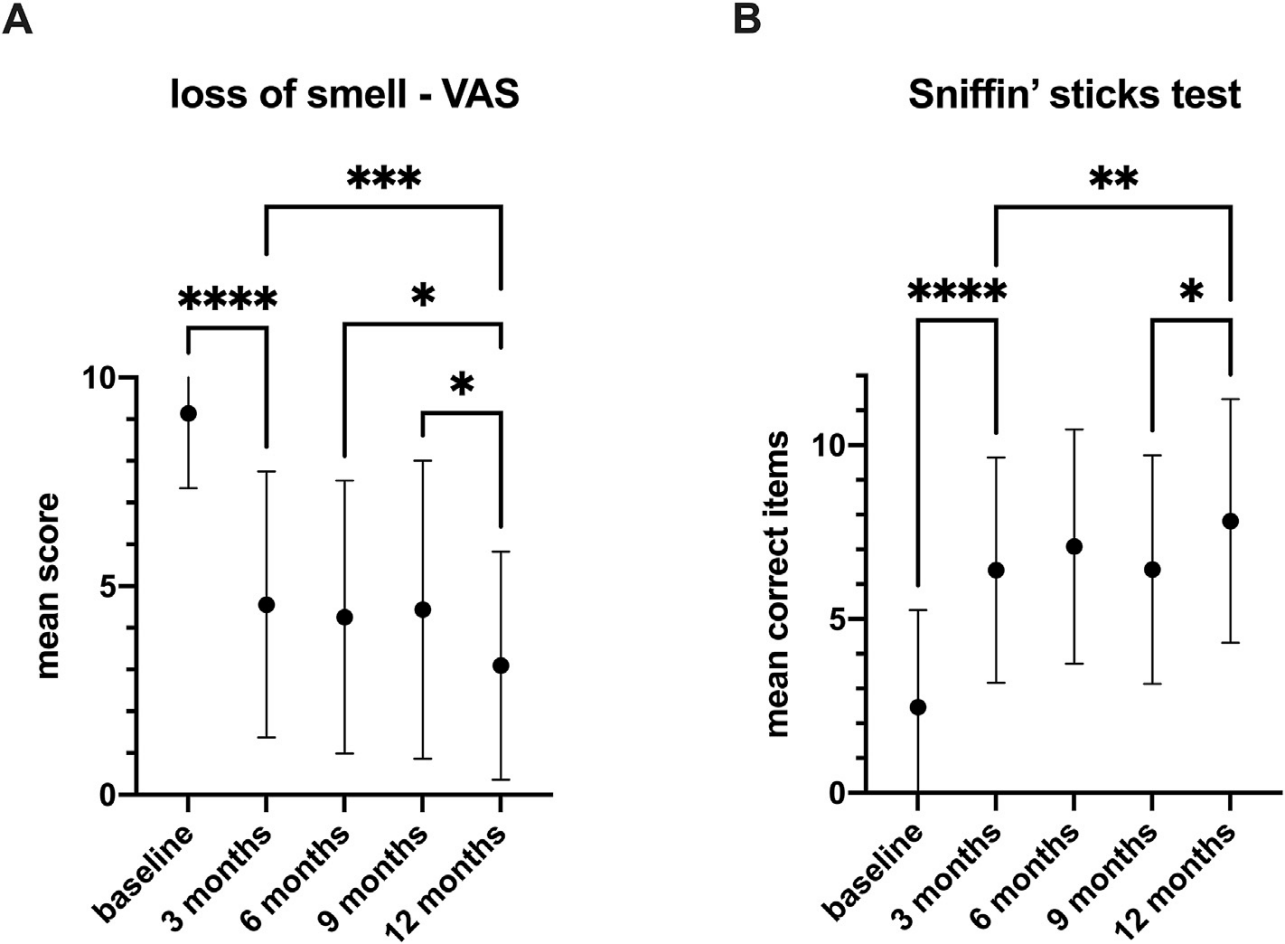
**A)** Perceived impairment of loss of smell scored on a VAS from 0 to 10 before and at 3,6,9 and 12 months after initiation of the dupilumab therapy. Mean values (dots) with standard deviation. ∗∗∗∗p < 0.0001; ∗∗∗p = 0.0005; ∗p < 0.05; **B)** Correct identified odors in the Sniffin’ Sticks 12 identification test on given timepoints of treatment. Mean values (dots) with standard deviation. ∗∗∗∗p < 0.0001; ∗∗p = 0.007; ∗p < 0.05.

The mean value of correctly identified odors in the Sniffin’ Sticks test before treatment improved significantly already after 3 months of treatment from 2.47 ± 2.78 to 6.41 ± 3.24 (p < 0.0001), and it continued to improve significantly to 7.82 ± 3.51 after 12 months (p = 0,007). The VAS scores and mean values of correctly identified odors are shown in Fig. 2.

#### Allergy and comorbid asthma

Given that CRSwNP patients often have concomitant asthma and/or allergies,^19^ patients were screened for these comorbidities prior to initiation of the dupilumab therapy. A total of 96.3% of the patient population had comorbidities with 61 (75.31%) patients having a diagnosed asthma and 24 (29.63%) patients having intermittent allergic rhinitis. Eighteen (22.22%) patients were diagnosed with persistent allergic rhinitis. Comorbid asthma was monitored with the GINA score, which captures symptom control from asthma-related symptom frequency, night waking and activity limitation, and, for patients using short-acting β2-agonist (SABA) reliever, the frequency of SABA use with simple yes or no questions. Here, a lower score indicates that asthma is better controlled. At the pretreatment timepoint, the mean score in patients with comorbid asthma was 2.7 ± 1.07. After 3 months of treatment, it decreased significantly to 1.68 ± 1.25 (p < <0.0001) and it continued to decrease to 1.09 ± 1.11 at the 12-month timepoint (p < 0.05). The course of the mean values over the survey period is shown in Fig. 1D.

#### Blood Eosinophils and IgE level

**Fig. 3:**
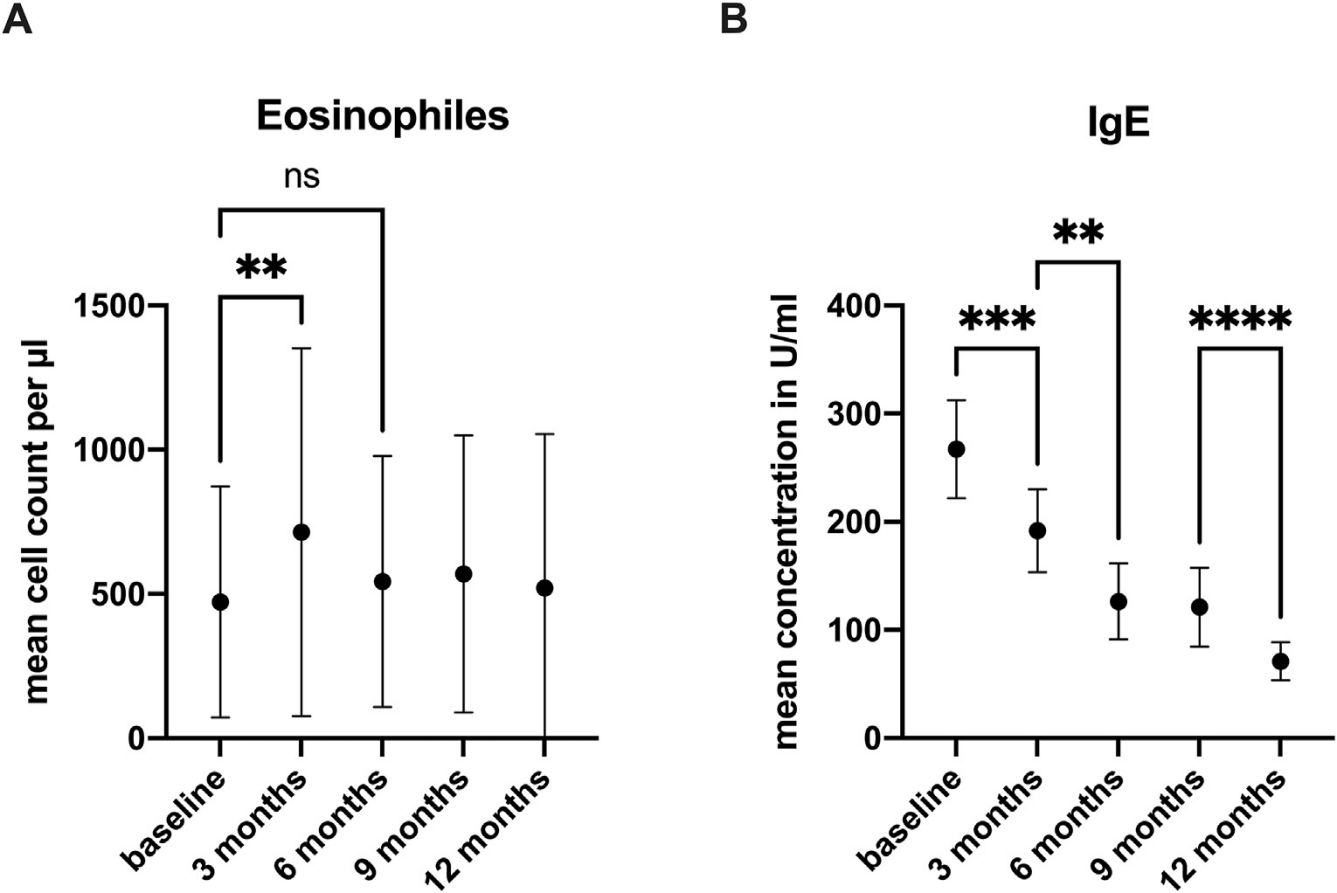
**A)** Means of total blood eosinophil counts before and at 3,6,9, and 12 months after initiation of the dupilumab therapy. Mean values (dots) with standard deviation. ∗∗p < 0.006; ns = not significant. **B)** IgE plasma levels before and at 3,6,9 and 12 months after initiation of the dupilumab therapy. Mean values (dots) with standard deviation. ∗∗p = 0.003; ∗∗∗p = 0.0004; ∗∗∗∗p = <0.0001.

At each timepoint, total blood eosinophil counts and IgE plasma levels were measured. The mean eosinophil count increased at the three-month timepoint from 472.5 ± 401.2 pretreatment to 714.5 ± 637.1 (p = 0.006) but decreased again to the initial level at the six-month timepoint (543.0 ± 434.7). The blood eosinophil level remained constant for the rest of the period examined (Fig. 3A).

IgE plasma levels decreased continuously over the observation period from a mean value of 267.2 ± 405.6 initially to 191.8 ± 336.1 after 3 months (p = 0.0004), 126.3 ± 245.9 (p = 0.003) after 6 months and finally to 70.92 ± 128 (p < 0.0001) after 12 months of therapy. Means with standard deviations are shown in Fig. 3B.

#### Safety/adverse events

Overall, several adverse events occurred in our cohort, leading to treatment discontinuation in 7 cases, with 1 case of hypereosinophilic syndrome. The patient with the hypereosinophilic syndrome reported increased difficulty breathing, abdominal spasms, fever, and increasing pain in the joints on the evening of the initial administration of dupilumab. With increasing symptoms, especially a worsening of the comorbid asthma, the patient was admitted to the hospital and had to be treated in the intensive care unit. Blood eosinophil counts showed an increased eosinophil percentage from an initial 9.6%–42%.

In total, 22 adverse events during treatment were reported. Local reactions, such as local erythema, itching or wheal at the injection site, arthralgia, and palpitations, were the most common (Table 2). Symptoms such as dizziness and nausea a few hours after the application of dupilumab, a generalized skin rash, the feeling of muscle soreness and joint stiffness and elevated body temperature were also reported by patients. One patient experienced loss of hair. Side effects that led to discontinuation of therapy included pain in the joints, dizziness, nausea, fever, loss of hair, and dry eyes.

**Table 2:** Adverse event during one year of treatment with Dupilumab

Adverse events (n)	22
Hypereosinophilic syndrome	1
Most common	
Local erythema	5
itching at injection site	3
Wheal at injection site	4
Arthralgia	8
Palpitation	2
Fatigue	2
Feeling of dry eyes	2
Others	7
Adverse events leading to treatment discontinuation	7

## Discussion

Dupilumab is a monoclonal antibody that interferes with type 2 inflammation, which is predominant in patients with CRSwNP. In the approval for other type 2 diseases, such as atopic dermatitis and severe or uncontrolled bronchial asthma, the efficacy of dupilumab has already been confirmed in several studies and meta-analyses.^20^^,^^21^ Significant improvement in patients' symptoms in phase III trials led to approval as an add-on therapy to intranasal corticosteroids in patients with CRSwNP by the European Medical Agency and German health authorities. However, since the strict rules of controlled trials do not allow the inclusion of the majority of patients who are treated by physicians in everyday life, the study populations and findings do not always compare well with patient populations treated under real-life conditions. With our prospective observational study of patients with severe uncontrolled CRSwNP, we showed the effectiveness and safety of dupilumab under real-life conditions over a one-year treatment period. In a Cochrane review with the aim of assessing the effects of biologics for the treatment of chronic rhinosinusitis, Ching et al found that almost all of the participants in the included studies had nasal polyps (99.8%) and that all were using topical nasal steroids for their chronic rhinosinusitis symptoms. In these patients, dupilumab improved disease-specific HRQL compared to placebo, and it likely also resulted in a reduction in disease severity.^22^

The significant improvement of disease-specific HRQL was demonstrated in the population of the SINUS-52 trial, where the SNOT-22 total scores decreased by 60% from the initial 50.16 to 19.71 after 52 weeks of treatment, and it was also found in our cohort with a similar reduction of the initial score of 59%. The rather conservative formulated statement that the therapy likely also leads to a reduction in the severity of the disease was affirmed by our results. Similar results in the improvement of HRQL were also observed in a per-protocol analysis of a smaller cohort in a six-month follow-up period,^23^ in a retrospective single center analysis with a cohort of 40 patients^24^ and an observational prospective study with 80 patients and follow up period of 16 weeks.^25^ Van der Lans also could show in a cohort of 24 patients with a follow-up of 48 weeks under real-life conditions an improvement in the SNOT22 total scores from initial 52.4 to 16.8.^26^ The NPS was significantly reduced in our cohort, and the perceived nasal congestion was also noticeably reduced within the first 3 months and remained at a low level over the observation period. The reduction of the NPS in our cohort was even more prominent after one year (73.45%) than the reduction after 52 weeks in the SINUS-52 trial, where a 41.52% reduction in NPS was reported. Ottaviano et al reported a 63.64% decrease in NPS after 1 year in a cohort of 47 patients, which is between our observation and the results of the phase III trials.^27^ Minagawa et al even found a 83% reduction in NPS in a cohort of 23 patients after 1 year of treatment.^28^

Although perceived nasal congestion was assessed with a different tool in the SINUS-52 study, a substantial reduction in this burden was demonstrated in both the SINUS-52-study cohort (58% reduction in NCS) and in our cohort (70% reduction in NC VAS) as in similar studies mentioned above .^24^^,^^27^ Loss of smell is one of the most important and difficult-to-treat symptoms for patients with CRSwNP.^29^ Bachert et al demonstrated rapid improvements in sense of smell. Differences versus placebo were evident by day 3 for daily patient-reported LoS and at the first assessments for UPSIT at week 2 of treatment.^29^ The mean change in the UPSIT in patients treated with subcutaneous dupilumab 300 mg every 2 weeks was 9.53 at week 52. Since the UPSIT is less common in Germany, we tested olfactory ability in our cohort with the routinely used Sniffin’ Sticks test.

In our cohort, similar to the data reported in the randomized controlled trials and other observational studies,^24^, ^25^, ^26^, ^27^, ^28^ there was a substantial improvement in olfactory ability in both objective testing and subjective impairment assessed by VAS.

The improvement in the sense of smell described by Mullol^29^ after only a few days of therapy is consistent with numerous patient statements from our cohort, although this was not systematically recorded at such an early stage in our study. The rapid improvement supports the theory that the loss of olfaction is not only due to a conductive component caused by nasal polyps as well as due to inflammation of the mucosa in the olfactory cleft.^30^^,^^31^ Since dupilumab is also approved for the treatment of other type 2 inflammatory diseases, such as atopic dermatitis and bronchial asthma, it is not surprising that asthma symptoms also improved in patients with comorbid asthma. This could be demonstrated by the significant reduction in the GINA score in our cohort as well as by other authors.^24^^,^^26^, ^27^, ^28^

As in the SINUS-52 study, at each sampling time point serum total IgE was measured in our cohort. We also recorded a constant decrease in concentrations of serum total IgE over the treatment period that was similar in the degree of reduction seen in the SINUS-52 trial (73.46% vs. 72.75% reduction). Total blood eosinophil counts (BEC) were also recorded at each time point and were comparable to the baseline values recorded in the SINUS study (472.5 vs. 450 cells/μl). Both SINUS studies reported a transient, but not significant, increase in mean BEC in patients treated with dupilumab, with a return to baseline levels by the end of the treatment period.^7^ IL-4 primes the vessel wall for extravasation of eosinophils through induction of vascular cell adhesion molecule (VCAM-1) and intercellular adhesion molecule (ICAM-1).^32^ The transient increase in BEC may be explained by blocking the VCAM-1 and ICAM-1 induction and by the hypothesis that dupilumab blocks eosinophil tissue migration by inhibiting the production of eotaxin-1 to 3 mediated by IL-4 and IL-13, but not the production of eosinophils or egress from bone marrow.^33^^,^^34^ Unlike the nonsignificant increase in BEC in the SINUS trials and other observational studies,^24^^,^^25^^,^^27^^,^^28^ in our population, we observed a significant increase in eosinophils at the 3-month timepoint. This has also been described by other authors who have used and evaluated dupilumab in other indications under real-word conditions.^35^ The observed normalization of BEC to baseline levels at the 6-month time point could be explained by a general reduction of the inflammatory response and thus reduction in production and release of eosinophils. Even though the eosinophilia had already returned to baseline values by the six-month measurement time point, the increase in eosinophils in our cohort led to a hypereosinophilic syndrome (BEC >1500 per μl for at least 6 month with organ system involvement^36^) in one patient and to discontinuation of the dupilumab therapy. This 25-year-old female patient presented with uncontrolled severe CRSwNP with comorbid asthma and an initial blood eosinophil count of 630 eosinophils per μl. There was no history of hypereosinophilia and no other clinical findings that would have suggested a dramatic increase in eosinophils during dupilumab therapy. Fourteen days after the first dupilumab injection, the patient presented with increasing cough accompanied by a feeling of shortness of breath and severe limb, joint, and muscle pain at another university hospital. The BEC was 7550 per μl. With additional elevated anti-MPO-ANCA and elevated Troponin T, the patient was admitted to the ICU and diagnosed with eosinophilic granulomatosis with polyangiitis (EGPA). The patient initially received a glucocorticoid therapy and was able to leave the hospital after one month of therapy with a long-term treatment of methotrexate and mepolizumab. Hypereosinophilic syndrome has also been reported in other case series in the context of dupilumab application in patients with severe asthma.^37^ Hypereosinophilic syndrome therefore appears to be a rare, albeit serious, side effect that should be considered when clinical signs are present. Of the 81 patients receiving therapy, 10 patients were classified as poor or nonresponders after one year of therapy according to the criteria proposed by the EUROFEA expert board meeting of 2021.^38^ Aiming to obtain a clinical indicator for poor or nonresponders, we examined the baseline characteristics of the cohort separated into responders and poor/nonresponders. No differences were found in baseline characteristics between nonresponders and those who responded well to therapy (data not shown). The lack of a control group and a possible selection bias due to recruitment in a tertiary referral center are limitations of this study. Further studies with patients who meet the indication criteria for therapy with biologics but are not as severely affected would provide more insight in the effectiveness of dupilumab under real-world conditions.

## Conclusion

If the indication for therapy with dupilumab in patients with CRSwNP is made according to the current recommendations of the EPOS 2022, the treated population in real-life is very similar to the group of patients included in the phase 3 trials, and the expected treatment effects are quite comparable. Dupilumab may show effectiveness and safety in the treatment of CRSwNP under real-world conditions. Systemic biomarkers or clinical parameters to predict a treatment response are currently missing. Further evaluation of new local and systemic biomarkers to better predict the treatment response is therefore necessary.

## Abbreviations

BEC, Total blood eosinophil counts; CRSsNP, chronic rhinosinusitis without nasal polyps; CRSwNP, chronic rhinosinusitis with nasal polyps; EPOS 2020, European position paper on rhinosinusitis and nasal polyps 2020; GINA Score, Global initiative for asthma score; HRQL, health-related quality of life; IL, Interleukin; NPS, nasal polyp score; SABA, short-acting β2-agonist; SNOT-22, Sino-Nasal Outcome Test-22; VAS, Visual Analog Scale.

## Funding

No funding.

## Availability of data and materials

Raw data were generated at the University hospital Tübingen; Ear Nose and Throat Department. The data that support the findings of this study are available from the corresponding author, upon reasonable request.

## Author contributions

M. M. Sailer, F. Capitani, C. van Schaik, S. Becker, and T. Albrecht made substantial contributions to data acquisition and were involved in patient management and organization. T. Albrecht analyzed the data and drafted the manuscript. H. Löwenheim was involved in critical revision of the manuscript. S. Becker designed the study, helped interpret the data, and reviewed the manuscript.

## Ethics statement

The research protocol for this prospective study was approved by the institutional ethics committee (University of Tübingen 873/2018BO2).

## Declaration of competing interest

None of the authors declares a competing interest.

## Submission declaration

Each of the authors confirms that this manuscript has not been previously published and is not currently under consideration by any other journal. Additionally, all of the authors have approved the contents of this paper and have agreed to the submission policies.
